# Editorial

**Published:** 1900-06-15

**Authors:** 


### EDITORIAL.

In this number we present to our readers obituary notices of two men who have had much to do with shaping the destiny of our profession not only in the localities where they lived, but throughout the entire country.

Dr. Geo. H. Cushing has had a long and useful life of professional labor as a practitioner, in which capacity he always endeavored to serve his patients with the highest order of technical skill. He was an ardent lover of his profession, and allied himself with every organization or movement which had for its object the betterment of his professional brethren. As a teacher in dental colleges he was eminently successful, and was held in the highest esteem by his confreres on the faculty and his students. His work for the National organization was of the same kind, and he gave the best years of his life to perfecting executive details of the organizations with which he was connected. He will be greatly missed, but the memory of his life and personality is a precious legacy.

Dr. Theo. Menges, who died June 1st, was in the full vigor of manhood, but died after a brief illness of appendicitis.

Probably no man connected with dental educational affairs in this country could have been called away with more loss to general educational interests at this time than Dr. Menges. He was not only the promoter and executive officer of one of our best and most largely attended dental schools, but was intensely interested in general professional educational matters. As Chairman of the Executive Committee of the National Association of Dental Faculties, he was aggressively doing all in his power to elevate educational standards. During the past winter he made an extensive visit to the dental schools of the country, to acquaint himself with the actual conditions under which the various schools were working, so that in his official capacity he would be able to intelligently act on all matters which might come before his committee. This will indicate somewhat the intense earnestness of the man, and explain in measure the cause for the great growth of the school of which he was the head. That such a man has been taken from us at this time, when his work is so much needed, is indeed a great loss.

The readers of this journal have also met a great loss in his death. He owned the DENTAL REGISTER, having purchased it last fall from Dr. Taft, and it was his plan, beginning with the coming year, to put his energy and money into it, with the intention of making it the leading dental publication of the world. Had his plans and hopes materialized, dental literature would have been richer and some radical changes made in the manner of conducting dental periodicals.

His life, although untimely cut off, was long enough to teach us that untiring energy and consecrated devotion can overcome the most formidable obstacles, and while mourning his loss, if inspired by his noble and generous motives, we may take up his task and complete his unfinished work.

